# Glaucoma
*A paper read to Bristol Medico-Chirurgical Society.


**Published:** 1972-01

**Authors:** C. A. Brown

**Affiliations:** Consultant Ophthalmologist, United Bristol Hospitals and Southmead Hospital


### Glaucoma *

**C. A. Brown, M.D., F.R.C.S.**

Consultant Ophthalmologist, United Bristol Hospitals and Southmead Hospital

The word glaucoma comes from the Greek word meaning "sea-green" because of the greenish appearance of the iris and pupil in an acute attack when seen through the oedematous cornea. It was known in the time of Hippocrates (460—360 B.C.) as a cause of one type of blindness. In the time of Galen (130—200 A.D.) the rise in intraocular pressure in glaucoma was recognised. In 1622 Banister first described absolute glaucoma as a very high tension, no perception of light and dilated pupil, but it was not till 1830 that William Mackenzie, the great Scottish clinician, in his book "Treatise on the Diseases of the Eye" finally separated glaucoma from cataract as different causes of blindness.

Glaucoma is now a relatively common eye condition. The incidence of primary glaucoma has been reported in several surveys (South Wales, Birmingham, Bedford) carried out from 1963-6 to be about 0.5% of the general population: e.g. In South Wales among 4,600 people, 24 had chronic glaucoma, 7 of these not previously diagnosed.

### What is Glaucoma?

Glaucoma is defined as a condition in which the intraocular pressure is raised above normal. This is true of all cases of acute and most cases of chronic, and also includes congenital and secondary glaucoma. This paper deals with acute and chronic primary glaucoma only. The circulation of aqueous is from the ciliary processes through the posterior into the anterior chamber and out through the trabecular meshwork and the canal of Schlemm.

In primary glaucoma the angle may be closed, leading to acute closed-angle glaucoma. Or it may be open, the condition then being chronic open-angle glaucoma. The mechanical theory of closed-angle glaucoma is well established (dilated pupil, crowded angle, angle closure, iris bombe, further closure). The cause of open-angle glaucoma is not so clear. It is widely thought to be due to sclerosis in the drainage channels from the anterior chamber, but Maurice Langham of Baltimore challenges this. He suggests that the increased intraocular pressure in chronic simple glaucoma is due to a breakdown in the regulating capacity of the eye which is controlled by adrenergic mechanisms (alpha-receptors through norepinephrine help outflow and beta-receptors through isoprenaline decrease aqueous formation). Thus this adrenergic mechanism controls the uveal blood flow, aqueous formation and aqueous outflow.

It is said that we have lost 50% of our regulating capacity by the age of 40. So there are two factors which may be responsible for chronic open-angle glaucoma—a sclerosis in the trabecular meshwork and an alteration in the vascular innervation of the anterior segment. To make matters more complicated, we find cases with all the clinical features of chronic glaucoma but with normal intraocular pressure—so-called "low tension glaucoma". These are due not to an increased intraocular pressure but to a decreased blood flow in the choroidal vessels round the disc. In rhesus and cynomolgus monkeys, experimental occlusion of a branch of the posterior ciliary artery damages part of the optic nerve and causes a nerve-fibre-bundle defect similar to a glaucoma defect. In both cases, fluorescein angiography will show the lack of fluorescence of the glaucomatous disc, due to occlusion of the vessels supplying it. We come then to a new definition of glaucoma—a disease in which the normal balance between the intraocular pressure and the blood supply of the optic nerve is disturbed, either from an increased intraocular pressure obliterating the circulation at the nerve-head, or a decreased blood flow from sclerosis of the vessels themselves ("low tension glaucoma"). In either case the result is an atrophy of the optic nerve.

### TYPES OF PRIMARY GLAUCOMA

As I have already indicated, there are two quite separate types:—

(a) Acute closed-angle glaucoma
(b) Chronic open-angle glaucoma

In 33 randomly selected cases of glaucoma in 1965 that came to operation, 20 were chronic simple glaucoma and 5 were acute. Both types occur in the second half of life, i.e., after 50.

### ACUTE CLOSED-ANGLE GLAUCOMA

(1) Causes (a) *Predisposing*

- (i) Small hypermetropic eye
- (ii) Shallow anterior chamber
- (iii) Narrow angle

(b) *Exciting*

Dilatation of the pupil leads to a crowding of the narrow angle by the iris and consequent obstruction of the outflow of aqueous. The cause may be physical or emotional.

*Physical* causes include dilatation of the pupil by drops or by systemic treatment. The latter includes the belladonna group of antispasmodics and also a number of synthetic drugs that are anticholinergic and have atropine-like effects, such as those in Parkinsonism (artane), some antidepressants of the tricyclic group (Tryptiline, Imipramine) or some tranquillisers of the Chlorpromazine group. All these

*A paper read to Bristol Medico-Chirurgical Society.*

cause a dry mouth and may cause poor accommodation and dilate the pupil. They have no effect on open-angle glaucoma.

*Emotional* causes include stress of any kind. They can be illustrated by the family row when an incensed wife threw a kipper at the husband's head. He developed acute glaucoma in the eye the kipper just missed! Or the middle-aged clerk who took an hour off from the office to see a Boris Karloff film. He gave himself three provocative tests at once—an hour in the dark, jugular compression from a tight collar and the emotional stress of the horror film. The personality of the patient is important—overanxious and excitable people are more liable than placid people to develop acute attacks.

**Clinical Features**

The first symptom is a transient halo occurring on several occasions in the previous few months. The haloes take the form of rainbow rings round lights in the dark.

The acute attack starts with severe pain in the eye, often with vomiting and rapid loss of vision, often within an hour. On examination the patient has a very red eye, often dark red. The cornea is hazy (from oedema), the anterior chamber is shallow in both eyes i.e. the iris is near the surface of the eye. The pupil is dilated and inactive, the iris and pupil, often dimly seen, may be greenish (glaucoma) and the iris pattern is dull. The tension is very high, even stony hard, to the finger test and on the tonometer.

**The differential diagnosis** is important:—

(1) The eye is inflamed in acute conjunctivitis but the cornea, iris and pupil are always normal.
(2) Corneal ulceration will show up as a local grey area and with a drop of fluorescein as a green stain.
(3) Acute iritis is most likely to cause confusion. Here the vision is only slightly impaired, the cornea is clear, the tension is normal.

### TREATMENT

**Medical** (1) Gutt. Pilocarpine 4% and Eserine 1% intensively every 5 minutes for 1 hour, and then in decreasing frequency to hourly, and hourly to opposite eye.
(2) *a.* Diamox 500 mg. intravenously,
or *b.* Glycerol orally — but vomiting is a contra-indication.
or *c.* Urea 30% as an intravenous drip can also be given.

**Surgical**

If the tension is still up after 3 hours and the cornea is still hazy (rare now) then an iridectomy, with or without iris inclusion, must be performed. There is a division of opinion here as to the type of iridectomy. Some say peripheral iridectomy is enough if the angle is 75% open, but even if it can be seen, the iris is always in apposition to the cornea and so it is safer to do an iris inclusion. If only a peripheral iridectomy is done there is a risk that the patient will again develop (or still have) glaucoma. A prophylactic peripheral iridectomy should be done on the other eye.

**One example:**—Some years ago a very nervous lady was admitted to Southmead Hospital with acute abdominal pain, vomiting and abdominal tenderness. She also complained of a painful right eye. Mr. M. G. Wilson asked me to see her and I found she had acute glaucoma (hazy cornea, shallow anterior chamber, dilated pupil, tension + + +) I asked Mr. Wilson if he was sure she had an acute abdomen, as the eye condition could be the cause of her vomiting. Mr. Wilson was sure and wanted to operate straight away. I was also sure, and so we decided to operate together. And we were both right! She had a ruptured appendix and acute glaucoma simultaneously. The anxiety caused by the acute appendicitis precipitated an attack of acute glaucoma—a very rare occurrence. Since then she has had an iris inclusion on her second eye and is still attending for supervision.

Last month I also had a patient who had been seen by a general surgeon for a year for intermittent vomiting. We found she had subacute closed-angle glaucoma in both eyes, and operation on her eyes cured her vomiting.

### CHRONIC (OPEN-ANGLE) GLAUCOMA

Here we are dealing with a much more insidious disease affecting the middle-aged or elderly. There is no pain, no inflammation, no apparent loss of vision till perhaps the disease is far advanced. The ocular pressure in both eyes rises slowly and there are no symptoms till late in the disease.

**Pathogenesis**

(a) **Mechanical factors.** The angle is open (wide, medium or narrow). The aqueous is secreted normally from the ciliary body but its drainage is defective. This is thought to be due to sclerosis in the trabecular meshwork and/or sclerosis in the draining network from the canal of Schlemm transclerally into the episcleral venous system and so back into the orbit. There is, however, no association with vascular hypertension but there is some local ocular vascular abnormality of a sclerotic nature.

(b) Another factor is concerned with vascular control of intraocular pressure through the regulating capacity of the eye, i.e. glaucoma may be a neurovascular disease. Adrenergic mechanisms come in here, as mentioned earlier in this paper.

(c) The disease can also be induced by local steroid drops or ointment to the eye.The dexamethasone group are responsible (Predsol, Betnesol), while the hydrocortisone group are safe. For example, I recently saw a patient whose blepharitis had been treated with Betnesol N ointment by her general practitioner and she had developed chronic glaucoma. The glaucoma cleared up on stopping the Betnesol N. The risk of glaucoma from prolonged systemic steroids is less but has been reported.

**Symptoms and Signs**

There are no symptoms till the disease is advanced, when visual loss will be noticed—perhaps loss of all useful vision in one eye. Sometimes the patients are too afraid to come and they wait till one eye is blind. The disease is usually detected by an optician at the time of refraction—he notices cupping of discs and may find a field defect. Sometimes the disease is found by examination of other members of the family of a glaucoma patient. If someone in your family has glaucoma you are ten times more likely to develop it than otherwise.

**Signs**

(1) The most important sign is pathological cupping of the disc. The pathological cup fills the whole disc whereas the physiological cup is central only. In the early stage there may be slight pallor of the disc only. The difficulty arises when the large physiological cup merges into an early pathological one.

(2) **Field defect.** This is tested by the Bjerrum screen or the Friedmann visual field analyser. The defects may be classified as follows:—

**Early**

(i) Arcuate scotoma (arching from the blind spot over or under the fixation point),
(ii) Baring of the blind spot to the periphery.
(iii) Upper nasal loss.

**Later**

Increased defects encroach towards the fixation point and so lead on to blindness.

(3) **Intraocular Pressure**

(a) Is measured by the **tonometer,** Schiotz (indentation method) or Applanation (corneal flattening method),
The ocular pressure may show diurnal variations —normal at visit to out-patients but above normal at 6 a.m. or 10 p.m. Hence there is real value in phasing.

(b) **Outflow facility** is measured by tonography. This is a test to record the amount of softening of the eye under continuous tonometer pressure for four minutes.

(c) **Provocative tests**—water drinking is best (1 litre on empty stomach)

(4) **Gonioscopy** shows the state of the angle as seen through the Goldmann gonioscopy lens.

**Treatment.** This may be medical or surgical. The medical treatment consists of
Pilocarpine drops 2-4% } q.i.d. with or
Eserine $\frac{1}{4}$-1% } without
Diamox Tabs. 250 mg. t.i.d. and
Potassium Effervescent 0.5 grm. b.d.
Tosmilen or Phospholine Iodide drops are sometimes used.

**The choice of treatment** depends on several factors.

(1) The control of the glaucoma by medical treatment. If it is not controlled, then operation is required.

(2) **Age of the patient.**

In the younger age group, operation is usually chosen. In the elderly no operation is performed if it can possibly be avoided because of the risk of increasing an early cataract.

(3) **Personality of the patient**

If the patient is meticulous and conscientious, drops are probably satisfactory as long as the tension is controlled medically. If the patient is a happy-go-lucky type of person who is liable to forget follow-up appointments and to throw out the drop bottles with the rubbish, then operation is better.
A miotic regime may be interrupted if the patient goes to hospital for another disease—say a heart attack or acute retention of the urine. One of my patients did this. His drops were continued but his Diamox was forgotten. The result was loss of vision with high intraocular tension and corneal oedema.

(4) **Personality of Ophthalmologist.**

The more cautious policy is medical treatment when, if things go wrong, the loss of vision is slow and insidious. If operation goes wrong, the loss is more sudden and dramatic. On the other hand, if vision is being progressively lost under maximum medical treatment, then operation is essential. I feel it is hard to sentence a young person to a miotic life. The vision is often darker after miotic drops and spasm of accommodation causes blurred distance vision for the first hour after instillation. Mr. Savin, a London colleague, suggests that doctors are divided by patients into "Pooh-pooh men" and "Wind-uppers". A patient may be treated with miotics for many months and nothing happens. The doctor is then labelled as a "Windupper" (!). The patient thinks the ophthalmologist is a fuss-pot and those of baser metal suspect their three-monthly routine visits to be not unconnected with the ophthalmologist's fees!!

**Operation.**

There are several drainage operations done for chronic glaucoma. They all aim at creating a new drainage channel for the aqueous under a conjunctival flap. The usual ones are:—

(1) Corneoscleral trephine (circular trephine hole of 1.5 mm.)

(2) Scheie's operation (cautery to lips of linear incision).

(3) Iris inclusion operation.

(4) Sclerectomy (removal of a wedge of sclera from a linear incision).

Various other complicated operations have been devised, such as trabeculectomy, but have not proved to be as reliable as the older methods.

The sign of a successful drainage operation is the formation of a filtration bleb, a raised area of conjunctiva under which aqueous filters from the anterior chamber into the sub-conjunctival tissues and so back into the venous channels of the orbit. There must be no direct communication with the outside of the eye. If there is, complications like intraocular infection or loss of anterior chamber are very liable to occur. The operation is usually done under local anaesthetic, but a general anaesthetic may be used. Post-operatively the patient is given mydriatics, antibiotic and steroid drops and is allowed home on these drops on the eighth or ninth day. The only immediate post-operative complication is delayed reformation of the anterior chamber, and this can be dealt with by reforming it in theatre. The long-term complication is the formation of a cataract, but this is likely only in the elderly if lens opacities are already present at the time of operation.

Long-term follow-up shows that 75% of drainage operations are effective and that on average the patient loses one line on the Snellen chart, i.e. 6/5 to 6/6 or 6/6 to 6/9. This is, however, perfectly normal vision and is well worth the reward of good control of the glaucoma—better, I think, than the best that can be obtained with miotics.

(A film was then shown of a corneo-scleral trephine operation).

